# Supplementary material for: Environmental Microbial Contamination during Cystic Fibrosis Group-Based Psychotherapy
Source: Int J Environ Res Public Health. 2021 Jan 28;18(3):1142. doi: 10.3390/ijerph18031142 (PMC7908138; doi:10.3390/ijerph18031142)
Supplement: Supplementary file 1 [file ijerph-18-01142-s001.pdf]

Table S1. Bacterial species recovered through surface sampling before psychological interventions

Class	Genus	Species	n°CFU
**Actinobacteria**	*Corynebacterium**	*Corynebacterium ureicelerivorans*	1
	*Micrococcus**	*Micrococcus luteus*	1
**Bacilli**	*Bacillus***	*Bacillus benzoevorans*	1
		Bacillus cereus	1
		Bacillus endophyticus	2
		Bacillus jeotgali	1
	*Paenibacillus***	*Paenibacillus polymyxa*	2
	*Staphylococcus**	*Staphylococcus epidermidis*	2
**Gammaproteobacteria**	*Acinetobacter**	*Acinetobacter lwoffii*	2

Note.* human-derived bacteria; ** truly-environmental bacteria; CFU=Colony Forming Units calculated as CFU/cm^2

Table S2. Bacterial species recovered through air and surface sampling after psychological interventions

Class	Genus	Species	n°CFU
**Actinobacteria**	*Actinomyces**	*Actinomyces dentalis*	9
		Actinomyces oris	3
	*Agromyces***	*Agromyces hippuratus*	1
		Agromyces italicus	3
		Agromyces rhizospherae	1
	*Atrhrobacter***	*Arthrobacter arilaitensis*	6
		Arthrobacter castelli	1
		Arthrobacter crystallopoietes	2
		Arthrobacter gandavensis	3
		Arthrobacter gangotriensis	2
		Arthrobacter kerguelensis	2
		Arthrobacter oxydans	4
		Arthrobacter polychromogenes	22
		Arthrobacter protophormiae	2
		Arthrobacter psychrophenolicus	2
		Arthrobacter russicus	1
		Arthrobacter sulfonivorans	4
		Arthrobacter woluwensis	1
	*Cellulosimicrobium***	*Cellulosimicrobium cellulans*	11
	*Corynebacterium**	*Corynebacterium afermentans*	1
		Corynebacterium glucuronolyticum	16
		Corynebacterium jeikeium	18
		Corynebacterium mucifaciens	6
		Corynebacterium singulare	1
		Corynebacterium tuberculostearicum	13
		Corynebacterium ureicelerivorans	4
	*Cutibacterium***	*Cutibacterium acnes*	1

		Cutibacterium avidum	3
	*Dermabacter***	*Dermabacter hominis*	2
	*Kocuria***	*Kocuria palustris*	2
		Kocuria rhizophila	4
		Kocuria rosea	4
	*Microbacterium***	*Microbacterium aurantiacum*	3
		Microbacterium dextranolyticum	1
		Microbacterium laevaniformans	1
		Microbacterium liquefaciens	1
		Microbacterium maritypicum	1
		Microbacterium oleivorans	6
		Microbacterium paludicola	9
	*Micrococcus**	*Micrococcus luteus*	496
		Micrococcus lylae	1
	*Nocardia***	*Nocardia farcinica*	2
	*Rothia**	*Rothia dentocariosa*	6
		Rothia mucilaginosa	1
	*Rhodococcus***	*Rhodococcus equi*	3
		Rhodococcus rhodochrous	1
		Rhodococcus ruber	1
	*Streptomyces***	*Streptomyces avidinii*	4
		Streptomyces badius	3
		Streptomyces chartreusis	3
		Streptomyces griseus	1
		Streptomyces hirsutus	2
		Streptomyces phaechromogenes	3
		Streptomyces violaceoruber	5
**Alphaproteobacteria**	*Acidiphilium***	*Acidiphilium acidophilum*	1
	*Brevundimonas***	*Brevundimonas diminuta*	3
		Brevundimonas vesicularis	2
	*Paracoccus***	*Paracoccus versutus*	1
		Paracoccus yeei	5
	*Roseomonas***	*Roseomonas mucosa*	1
	*Sphingomonas***	*Sphingomonas sp.*	1
	*Sphingopyxis***	*Sphingopyxis terrae*	2
	*Starkeya***	*Starkeya novella*	1
**Bacilli**	*Aneurinibacillus***	*Aneurinibacillus migulanus*	1
	*Aerococcus***	*Aerococcus viridans*	1
	*Bacillus***	*Bacillus acidicola*	1
		Bacillus altitudinis	1
		Bacillus arsenicus	17
		Bacillus asahii	1
		Bacillus barbaricus	1
		Bacillus bataviensis	5
		Bacillus cellulosilyticus	1
		Bacillus cereus	39
		Bacillus circulans	1

	Bacillus cohnii	1
	Bacillus drentensis	1
	Bacillus firmus	40
	Bacillus flexus	3
	Bacillus galactosidilyticus	2
	Bacillus horneckiae	1
	Bacillus humi	1
	Bacillus hwajinpoensis	1
	Bacillus idriensis	1
	Bacillus indicus	2
	Bacillus jeotgali	4
	Bacillus licheniformis	33
	Bacillus marisflavi	3
	Bacillus megaterium	49
	Bacillus mojavensis	5
	Bacillus muralis	1
	Bacillus mycoides	2
	Bacillus niacini	10
	Bacillus novalis	3
	Bacillus odysseyi	2
	Bacillus psychrodurans	1
	Bacillus pumilus	5
	Bacillus safensis	1
	Bacillus simplex	22
	Bacillus siralis	1
	Bacillus sonorensis	1
	Bacillus subtilis	15
	Bacillus thuringiensis	2
*Brevibacillus***	*Brevibacillus parabrevis*	1
	Brevibacillus paucivorans	3
	Brevibacillus ravenspurgense	1
*Enterococcus***	*Enterococcus casseliflavus*	1
*Exiguobacterium***	*Exiguobacterium sp.*	7
	Exiguobacterium aurantiacum	8
*Lactobacillus**	*Lactobacillus amylotrophicus*	2
	Lactobacillus coleohominis	2
	Lactobacillus coryniformis	4
	Lactobacillus fuchuensis	12
	Lactobacillus gasseri	1
	Lactobacillus graminis	1
	Lactobacillus ingluviei	1
	Lactobacillus johnsonii	1
	Lactobacillus kefiri	2
	Lactobacillus oris	1
	Lactobacillus paracasei	4
	Lactobacillus paralimentarius	27
	Lactobacillus pentosus	4
	Lactobacillus sakei	6
*Listeria***	*Listeria innocua*	1

	*Macrococcus***	*Macrococcus caseolyticus*	1
	*Paenibacillus***	*Paenibacillus amylolyticus*	2
		Paenibacillus barengoltzii	2
		Paenibacillus Illinoisensis	1
		Paenibacillus lautus	100
		Paenibacillus massiliensis	1
		Paenibacillus mendelii	1
		Paenibacillus polymyxa	2
		Paenibacillus timonensis	1
		Paenibacillus xylanilyticus	1
	*Solibacillus***	*Solibacillus silvestris*	3
	*Staphylococcus**	*Staphylococcus arlettae*	2
		Staphylococcus aureus	8
		Staphylococcus capitis	48
		Staphylococcus carnosus	2
		Staphylococcus cohnii	13
		Staphylococcus epidermidis	288
		Staphylococcus haemolyticus	19
		Staphylococcus hominis	81
		Staphylococcus lugdunensis	5
		Staphylococcus pasteuri	1
		Staphylococcus pettenkoferi	1
		Staphylococcus saprophyticus	11
		Staphylococcus simiae	6
		Staphylococcus warneri	14
		Staphylococcus xylosus	18
	*Streptococcus**	*Streptococcus agalactiae*	1
		Streptococcus castoreus	4
		Streptococcus constellatus	3
		Streptococcus cristatus	1
		Streptococcus equi	1
		Streptococcus mitis	1
		Streptococcus oralis	7
		Streptococcus parasanguinis	10
		Streptococcus salivarius	2
		Streptococcus sanguinis	13
		Streptococcus suis	1
	*Virgibacillus***	*Virgibacillus pantothenticus*	1
	*Weissella***	*Weissella minor*	1
		Weissella viridescens	1
**Bacteroidia**	*Bacteroides***	*Bacteroides fragilis*	1
**Betaproteobacteria**	*Aromatoleum***	*Aromatoleum toluolicum*	1
	*Burkholderia***	*Burkholderia andropogonis*	1
		Burkholderia anthina	5
		Burkholderia metallica	1
	*Cupriavidus***	*Cupriavidus necator*	1
		Cupriavidus respiraculi	2
	*Neisseria**	*Neisseria flavescens*	3
**Clostridia**	*Clostridium***	*Clostridium beijerinckii*	7

		Clostridium cochlearium	5
		Clostridium innocuum	3
		Clostridium sordellii	3
**Flavobacteria**	*Chryseobacterium***	*Chryseobacterium joostei*	2
	*Elizabethkingia***	*Elizabethkingia meningoseptica*	1
	*Flavobacterium***	*Flavobacterium flevense*	1
**Gammaproteobacteria**	*Acinetobacter**	*Acinetobacter baumanii*	1
		Acinetobacter junii	1
		Acinetobacter lwoffii	32
		Acinetobacter pittii	3
		Acinetobacter radioresistens	1
	*Balneatrix***	*Balneatrix alpica*	1
	*Enterobacter**	*Enterobacter aerogenes*	3
		Enterobacter ludwigii	1
	*Escherichia***	*Escherichia vulneris*	1
	*Halomonas***	*Halomonas elongata*	1
	*Leclercia***	*Leclercia adecarboxylata*	1
	*Moraxella**	*Moraxella spp*	4
	*Pantoea**	*Pantoea agglomerans*	4
	*Pseudomonas***	*Pseudomonas abietaniphila*	2
		Pseudomonas balearica	2
		Pseudomonas flavescens	1
		Pseudomonas fulva	1
		Pseudomonas luteola	1
		Pseudomonas massiliensis	1
		Pseudomonas oryzihabitans	12
		Pseudomonas plecoglossicida	1
		Pseudomonas putida	4
		Pseudomonas rhodesiae	2
		Pseudomonas sp.	1
		Pseudomonas straminea	4
		Pseudomonas stutzeri	4
		Pseudomonas vancouverensis	3
		Pseudomonas xanthomarina	1
	*Raoultella ***	*Raoultella ornithinolytica*	1
	*Serratia***	*Serratia plymuthica*	1
	*Stenotrophomonas***	*Stenotrophomonas maltophilia*	4
**Sphingobacteria**	*Sphingobaterium***	*Sphingobacterium multivorum*	2
		Sphingobacterium spiritivorum	1
**Tissierellia**	*Tissierella***	*Tissierella praeacuta*	1

Note.* human-derived bacteria; ** truly-environmental bacteria; CFU=Colony Forming Units calculated as CFU/m²/h and CFU/cm²

Table S3 Bacterial species recovered through air and surface sampling after the first five psychological interventions

Class	Genus	Species	n°CFU
**Actinobacteria**	*Arthrobacter***	*Arthrobacter woluwensis*	1

	*Cellulosimicrobium***	*Cellulosimicrobium cellulans*	1
	*Kocuria***	*Kocuria rosea*	1
	*Microbacterium***	*Microbacterium paludicola*	5
	*Micrococcus**	*Micrococcus luteus*	79
	*Rhodococcus***	*Rhodococcus ruber*	1
	*Streptomyces***	*Streptomyces violaceoruber*	1
**Alphaproteobacteria**	*Paracoccus***	*Paracoccus yeei*	2
	*Sphingomonas***	*Sphingomonas spp.*	1
	*Starkeya***	*Starkeya novella*	1
**Bacilli**	*Aerococcus***	*Aerococcus viridans*	1
	*Bacillus***	*Bacillus acidicola*	1
		Bacillus arsenicus	8
		Bacillus barbaricus	1
		Bacillus bataviensis	2
		Bacillus cereus	20
		Bacillus drentensis	1
		Bacillus firmus	27
		Bacillus flexus	2
		Bacillus humi	1
		Bacillus licheniformis	18
		Bacillus marisflavi	2
		Bacillus megaterium	18
		Bacillus muralis	1
		Bacillus mycoides	1
		Bacillus niacini	1
		Bacillus pumilus	2
		Bacillus safensis	1
		Bacillus simplex	11
		Bacillus siralis	1
		Bacillus subtilis	4
	*Exiguobacterium***	*Exiguobacterium sp.*	2
		Exiguobacterium aurantiacum	2
	*Lactobacillus**	*Lactobacillus coleohominis*	2
		Lactobacillus ingluviei	1
	*Paenibacillus***	*Paenibacillus amylolyticus*	2
		Paenibacillus lautus	100
		Paenibacillus massiliensis	2
		Paenibacillus polymyxa	1
		Paenibacillus timonensis	1
		Paenibacillus xylanilyticus	1
	*Staphylococcus**	*Staphylococcus aureus*	1
		Staphylococcus capitis	6
		Staphylococcus epidermidis	16
		Staphylococcus haemolyticus	1
		Staphylococcus hominis	6
		Staphylococcus warneri	2
**Clostridia**	*Clostridium***	*Clostridium cochlearium*	2
		Clostridium innocuum	1
**Gammaproteobacteria**	*Acinetobacter**	*Acinetobacter lwoffii*	8

*Pantoea**	*Pantoea agglomerans*	3
*Pseudomonas***	*Pseudomonas abietaniphila*	1
	Pseudomonas balearica	2
	Pseudomonas oryzihabitans	1
	Pseudomonas stutzeri	2
*Stenotrophomonas***	*Stenotrophomonas maltophilia*	1

Note.* human-derived bacteria; ** truly-environmental bacteria; CFU=Colony Forming Units calculated as CFU/m^2/h and CFU/cm^2
